# Profiling the Extended Cleavage Specificity of the House Dust Mite Protease Allergens Der p 1, Der p 3 and Der p 6 for the Prediction of New Cell Surface Protein Substrates

**DOI:** 10.3390/ijms18071373

**Published:** 2017-06-27

**Authors:** Alain Jacquet, Vincenzo Campisi, Martyna Szpakowska, Marie-Eve Dumez, Moreno Galleni, Andy Chevigné

**Affiliations:** 1 Faculty of Medicine, Division of Research Affairs, Chulalongkorn University, 10330 Bangkok, Thailand; alain.J@chula.ac.th; 2 Department of Infection and Immunity, Luxembourg Institute of Health (LIH), 29, rue Henri Koch, L-4354 Esch-sur-Alzette, Luxembourg; vcampisi@live.be (V.C.); martyna.szpakowska@lih.lu (M.S.); marie-eve.x.dumez@gsk.com (M.-E.D.); 3 Laboratoire des Macromolécules Biologiques, Centre for Protein Engineering (CIP), University of Liège, 4000 Liège, Belgium; mgalleni@ulg.ac.be

**Keywords:** house dust mite, *Dermatophagoides pteronyssinus*, allergen, protease, phage display, cell surface proteome, phage substrate

## Abstract

House dust mite (HDM) protease allergens, through cleavages of critical surface proteins, drastically influence the initiation of the Th2 type immune responses. However, few human protein substrates for HDM proteases have been identified so far, mainly by applying time-consuming target-specific individual studies. Therefore, the identification of substrate repertoires for HDM proteases would represent an unprecedented key step toward a better understanding of the mechanism of HDM allergic response. In this study, phage display screenings using totally or partially randomized nonameric peptide substrate libraries were performed to characterize the extended substrate specificities (P_5_–P_4_′) of the HDM proteases Der p 1, Der p 3 and Der p 6. The bioinformatics interface PoPS (Prediction of Protease Specificity) was then applied to define the proteolytic specificity profile of each protease and to predict new protein substrates within the human cell surface proteome, with a special focus on immune receptors. Specificity profiling showed that the nature of residues in P_1_ but also downstream the cleavage sites (P′ positions) are important for effective cleavages by all three HDM proteases. Strikingly, Der p 1 and Der p 3 display partially overlapping specificities. Analysis with PoPS interface predicted 50 new targets for the HDM proteases, including 21 cell surface receptors whose extracellular domains are potentially cleaved by Der p 1, Der p 3 and/or Der p 6. Twelve protein substrate candidates were confirmed by phage ELISA (enzyme linked immunosorbent assay). This extensive study of the natural protein substrate specificities of the HDM protease allergens unveils new cell surface target receptors for a better understanding on the role of these proteases in the HDM allergic response and paves the way for the design of specific protease inhibitors for future anti-allergic treatments.

## 1. Introduction

House dust mites (HDMs) represent an important source of airborne allergens associated with various inflammatory diseases, such as allergic asthma, perennial rhinitis, conjunctivitis and atopic dermatitis [1]. Amongst the 20 allergens identified so far in the HDM species *Dermatophagoides pteronyssinus* (Available online: www.allergen.org), Der p 1, Der p 3 and Der p 6 display proteolytic activities [2]. Der p 1 is a papain-like cysteine protease, whereas Der p 3 and Der p 6 are serine proteases with tryptic and chymotryptic activities, respectively [2]. Notably, Der p 1 is not only the most abundant HDM allergen in house dust or in mite cultures but also a potent allergenic protein as more than 80% of the HDM allergic population develop high level of IgE specific to this protease [3,4]. Der p 1 has also been demonstrated to act as the activator of the precursors of Der p 3 and Der p 6 according to an uncommon activation cascade [5,6,7]. In contrast to Der p 1, little is known about the concentration of Der p 3 and Der p 6 in mite cultures and their IgE binding frequencies remain poorly characterized with IgE prevalence ranging from 10% to 50% for Der p 3 and around 40% for Der p 6 [8,9,10].

It is now well established that the proteolytic activities of HDM allergens drastically influence the development of the allergic response through different mechanisms [11], including: (I) the disruption of the epithelial barrier integrity through cleavages of the lung epithelium surfactant proteins SP-A, SP-D [12], the tight junction proteins occludin, zona occludens-1 (ZO-1) and cadherins [13]; (II) the activation of damage-associated molecular patterns (DAMPS) such as uric acid [14,15]; (III) the direct activation of protease-activated receptors (PARs) expressed on airway epithelial cells and keratinocytes [16,17,18]; (IV) the cleavage of immune receptors expressed by dendritic (CD40 and DC-SIGN) [19], B (CD23) [20] or T (CD25) [21,22] cells; and (V) the inactivation of protease inhibitors such as the α1-antitrypsin [23], the elastase-specific inhibitor (elafin) and secretory leukocyte protease inhibitor and homeostasis proteins [24,25].

Altogether, the cleavages of these cellular receptors and secreted proteins by the HDM proteases influence the initiation of allergic sensitization and may lead to the exacerbation of allergic inflammation by promoting a pro-Th2 environment and/or by downregulating the Th1/Treg differentiation [1,11,26]. It should be pointed out that so far the different human protein substrates identified for Der p 1, Der p 3 or Der p 6 were discovered essentially by individual and targeted studies [16,17,22,27] and that the identification of their complete repertoire of cellular substrates is still unachieved [19]. Notably, with the exception of the tight junction proteins, the airway epithelial cell surface receptor(s) targeted by Der p 1 remain curiously unknown [1,2].

**Figure 1:**
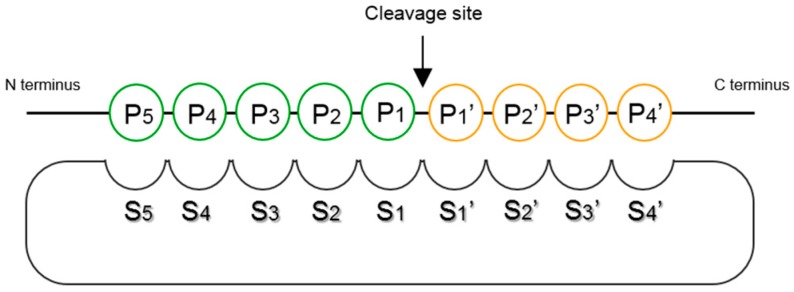
Representation of the interplay between a protease and a peptide substrate at the catalytic cleft. P/P′: substrate residues located upstream/downstream of the peptide cleavage site; S/S′: corresponding subsites at the protease active site [28].

The interplay between a defined protease and its corresponding substrates is mainly mediated by the structure of the active site cleft, which determines the type of residues compatible with the different substrate binding sites/pockets (subsites S1–5 and S1′–4′). Determining the substrate specificity of a protease consists in identifying consensus residues located upstream (P_5_–P_1_) and/or downstream (P_1_′–P_4_′) of the peptide cleavage site, which can be best accommodated by the corresponding subsites (Figure 1) [28].

The specificity of Der p 1 has previously been investigated using individual substrates and microarrays of fluorogenic tetrapeptides but was limited to positions P_4_–P_1_ [29,30]. The substrate specificity of Der p 1 was shown to be mainly dependent on the S2 subsite, displaying high preference for peptides with Alanine (Ala) in P_2_ position. The S_1_, S_3_ and S_4_ subsite specificities were less stringent with a preference for basic amino acids in P_1_ and P_3_ and aliphatic amino acids in P_4_ [29]. Little is known about the cleavage specificities of Der p 3 and Der p 6. Although our previous results confirmed that these two proteases display tryptic and chymotryptic activities with a preference for substrates with lysine/arginine and tyrosine/phenylalanine in P_1_, respectively, their P_5_–P_2_ and P_1_′–P_4_′ subsite specificities remain to be fully elucidated [5,7].

The present study aims at profiling the extended substrate specificities (P_5_–P_4_′) of the HDM protease allergens Der p 1, Der p 3 and Der p 6 by phage display technology and predicting, using the interface PoPS (Prediction of Protease Specificity), new potential substrates among the human cell surface proteome that could play a role in the HDM allergic response.

## 2. Results

### 2.1. Determination of Extended Der p 1, Der p 3 and Der p 6 Substrate Specificities

Three fully or partially randomized nonameric substrate libraries were displayed at the amino terminus of the coat protein pIII of M13 phage. The specificity of Der p 1 was evaluated using a library displaying random residues at positions P_5_ to P_4_′, including the residue P_1_ (X_4_-X-X_4_). Indeed, although previous studies using tetrapeptides indicated that Der p 1 shows a marked preference for an arginine or a lysine at P_1_, several cleavage sites identified on natural protein targets, such as CD23 or DC-SIGN, do not display these basic residues at this position. In addition, no clear consensus for positions P_5_ to P_4_′ could be inferred from the limited number of natural substrates identified so far, suggesting that the cleavage specificity of Der p 1 may be more complex than initially thought [19,20,22,27]. In contrast, given the clear sequence and activity resemblance of Der p 3 and Der p 6 with trypsin and chymotrypsin, requiring the presence at P_1_ of an arginine or a lysine and a tyrosine or a phenylalanine, respectively, the P_1_ residues in libraries used to determine the specificity of these two HDM proteases were fixed (X_4_-R/K-X_4_ and X_4_-Y/F-X_4_) (Table 1) [5,7,8,31]. Prior to their use in screening campaigns, the integrity of the displayed sequences and the diversity of amino acids at each position were confirmed (Figure S1).

The libraries X_4_-X-X_4_, X_4_-R/K-X_4_ and X_4_-Y/F-X_4_ were then screened to determine the cleavage specificity profiles of Der p 1, Der p 3 and Der p 6, respectively. For each protease, three selection rounds with increasing stringency (decreasing elution times with or without decreasing protease concentration) were performed to progressively select phages displaying the optimal sequences. Increasing enrichment rates from the first to the third selection round were observed for each screening campaign. The addition of Der p 1 to the library X_4_-X-X_4_ at selection rounds 1 (30 min with 80 nM Der p 1), 2 (27 min with 80 nM Der p 1) and 3 (24 min with 80 nM Der p 1) resulted in, respectively, 8, 9 and 10 times greater release of phages in comparison with the control experiment. Enrichment rates of 610, 810 and 980 were obtained with the library X_4_-R/K-X_4_ and Der p 3 for rounds 1 (30 min with 8 nM Der p 3), 2 (15 min with 2 nM Der p 3) and 3 (10 min with 1 nM Der p 3), respectively. Enrichment rates of 280, 320 and 480 were observed at selection rounds 1 (24 min with 6 nM Der p 6), 2 (21 min with 4 nM Der p 6) and 3 (18 min with 3 nM Der p 6), respectively, with the library X_4_-Y/F-X_4_ and Der p 6.

**Figure 2:**
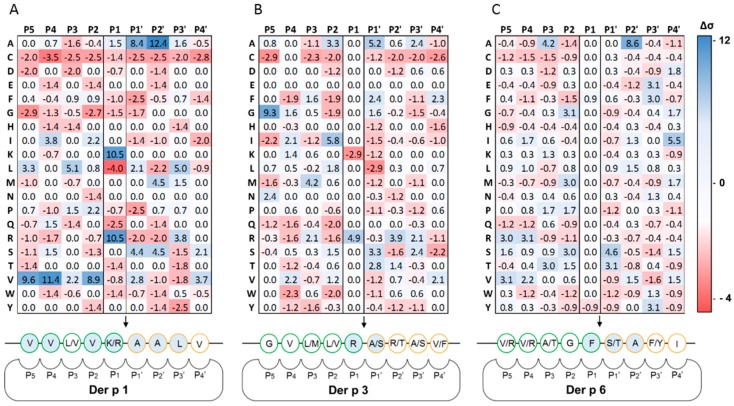
Substrate specificity profile of Der p 1, Der p 3 and Der p 6 for positions P_5_ to P_4_′. A to C: HDM protease specificity matrices based on standard deviations (∆σ) calculated for each amino acid at positions P_5_ to P_4_′ after three rounds of selection from libraries: (**A**) X_4_-X-X_4_ with Der p 1; (**B**) X_4_-R/K-X_4_ with Der p 3; and (**C**) X_4_-Y/F-X_4_ with Der p 6. Standard deviations (Δσ) account for the selection efficiencies of any amino acid at each position. Positive and negative Δσ correspond to scores attributed to positively and negatively selected amino acids, respectively. Der p 1 specificity relied on P_4_, P_2_, P_1_, P_1_′ and P_2_′ residues. Der p 3 was highly specific for positions P_1_ and P_1_′. Der p 6 cleavage properties were strongly affected by P_1_, P_1_′ and P_2_′ residues.

For each protease, 75 clones from the third selection round were randomly chosen for sequencing. Sequence alignments showed significant redundancies allowing to determine Der p 1, Der p 3 and Der p 6 cleavage patterns and to build the corresponding specificity models for P_5_–P_4_′ residues (Figure 2). Based on phage substrate selection campaigns, several features in the specificity of HDM proteases were highlighted.

**Figure 3:**
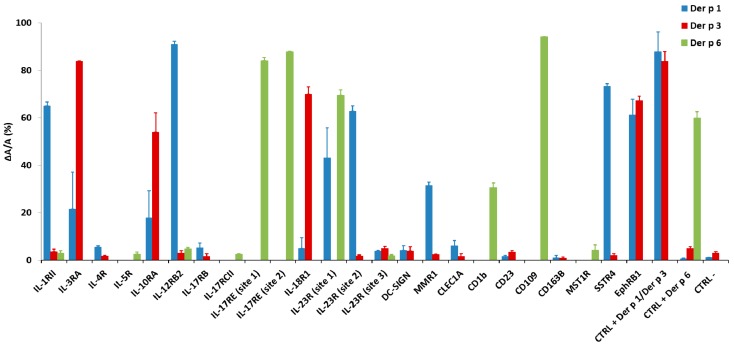
Confirmation of predicted cleavage site proteolysis by HDM proteases. Cleavage efficiencies of the predicted sites present at the 21 potential targets (ΔA/A) by Der p 1, Der p 3 and Der p 6 are represented in blue, red and green, respectively. Individual purified phages were either untreated (A) or treated (∆A) with Der p 1, Der p 3 or Der p 6 for 30 min at 37 °C before ELISA on Ni-NTA HisSorb plates. Data presented as mean of two independent experiments performed in duplicate ± standard error of the mean. Significance was calculated compared to negative control using a Mann-Whitney test (*p* value 0.05).

**Figure 4:**
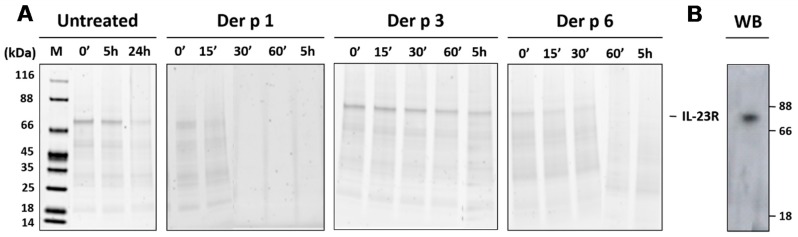
(**A**) SDS-PAGE and Western blot analyses of rIL-23R (10 nM) proteolysis by Der p 1 (2.5 nM), Der p 3 (0.25 nM) and Der p 6 (0.5 nM). Samples were analyzed after different incubation times. kDa: kiloDalton; M: molecular weight marker (Pierce Unstained Protein MW marker, Thermo Fisher Scientific, Rockford, USA); (**B**) Western blot analysis using a rabbit anti-human IL-23R (residues 62–75) monoclonal primary antibody and a HRP-conjugated monoclonal anti-rabbit IgG as secondary antibody.

Notably, Der p 1 was shown to have a strong preference for peptide substrates with Val residues located at P_5_ (Δσ = 9.6), P_4_ (Δσ = 11.4) and P_2_ (Δσ = 8.9). Lysine (Lys) or Arginine (Arg) were equivalently represented at P_1_ (Δσ = 10.5) and Alanine (Ala) residues were preferred at P_1_′ (Δσ = 8.4) and P_2_′ (Δσ = 12.4) (Figure 3A). Der p 3 and Der p 6 showed stringency in their substrate specificities at P_1_ and P_1_′. Der p 3 favored Glycine (Gly) at P_5_ (Δσ = 9.3), Arg residues at P_1_ (Δσ = 4.9) as only a few Lys residues were found at this position at the end of the selection (Δσ = −2.9) and showed a marked preference for Ala at P_1_′ (Δσ = 5.2) and (Figure 4B). Remarkably, for P_1_ and P_1_′ the specificity profile of Der p 3 partially overlaps with that of Der p 1 (Figure 3A,B). Der p 6 specificity was shown to be dependent on Ala residue at P_3_ (Δσ = 4.2) and P_2_′ (Δσ = 8.6) as well as Ile in P_4_′. Its specificity towards P_1_ residues was slightly higher for Phenylalanine (Phe) (Δσ = 0.9) than for Tyrosine (Tyr) (Δσ = −0.9), while at P_1_′ Serine (Ser) and Threonine (Thr) were preferred (Δσ of 4.6 and 3.1, respectively) (Figure 3C). For all three proteases, large aromatic residues, especially Tryptophan (Trp), were not well tolerated at positions P_4,_ P_2_ and P_1_′. Similarly, Cysteine (Cys) residues were also found to be less represented at almost all positions after selection with Der p 1, Der p 3 and Der p 6. Finally, Gly at positions P_5_ to P_1_′ had a severe negative impact on Der p 1 cleavage efficacy. Similar negative effect was observed for Proline (Pro) at position P_1_′ for the three proteases. Other minor positive and negative determinants of the HDM protease specificities were also detected but seemed to influence their cleavage properties to a lesser extent.

### 2.2. In Silico Prediction of Potential Protein Substrates within the Human Cell Surface Proteome

The standard deviation values (Δσ) calculated for each residue at each position (Figure 2) were taken into consideration to build specificity matrices for positions P_5_ to P_4_′ for Der p 1, Der p 3 and Der p 6. Individual specificity scores were normalized to the PoPS scale range (−5 to +5) (Figure S1) and used to predict potential targets within the human cell surface proteome using the Prediction of Protease Specificity (PoPS) bioinformatics tool with a search threshold score of 10. Predicted hits among the full human proteome were sorted out and cleaned. Hypothetical and predicted proteins found in the dataset were not taken into account. Among the 500 best hits, the 10% displaying at least one extracellular domain susceptible to proteolysis were selected (Table S1). Moreover, proteins directly or indirectly involved in immune mechanisms related to allergy and inflammation on the basis of experimental findings/literature were privileged. At the end of this selection process, 21 proteins containing at least one extracellular site susceptible to proteolysis and potentially related to the HDM allergic response were shortlisted (Table 2).

Among these 21 new potential targets for HDM proteases, 11 interleukin receptors, two carbohydrate-binding receptors (CD1b and MMR1) and five other receptors (CD109, CD163b, MST1R, SSTR4 and EphRB1) involved in immune pathways were predicted (Table 2). Interestingly, some of these targets were predicted to be specifically cleaved by one HDM protease only, while others by both Der p 1 and Der p 3, which is in agreement with their observed partially overlapping specificities. Noteworthy, among the 21 shortlisted hits predicted by PoPS, two targets, DC-SIGN and CD23, were previously shown to be cleaved effectively by Der p 1 [19,20].

### 2.3. Validation of the Predictions by ELISA Using Phage Peptide Substrates

To confirm that the predicted cleavage sequences present within the 21 potential novel targets can be efficiently recognized by a particular HDM protease, the sequences corresponding to the 24 predicted cleavage sites identified using PoPS were displayed on phage particles, incubated with Der p 1, Der p 3 or Der p 6 and analyzed by phage ELISA (enzyme linked immunosorbent assay). Peptide sequence LNARTNAS, an optimized mimic of proDer p 1 propeptide C-terminal activation site (LNAETNAC), was used as positive control for Der p 1 and Der p 3, whereas peptide sequence QPKWSYLDS corresponding to proDer p 6 internal propeptide cleavage site recognized by Der p 6 [5] was used as positive control for this protease. Sequence GGSGGSGGS was used as negative control for the three proteases. Eighty percent of phages displaying the positive control sequence LNARTNAS were efficiently cleaved by Der p 1 and Der p 3 and 60% of phages displaying QPKWSYLDS were cleaved by Der p 6. Less than 3% of the negative control phages were cleaved by the three HDM proteases.

Overall, about 90%, 50% and 100% of the predicted sequences for Der p 1, Der p 3 or Der p 6 were significantly cleaved in phage ELISA, respectively. For Der p 1, 12 out of the 13 sequences predicted were confirmed. Only CD163B was not confirmed for Der p 1. Additionally, three targets initially predicted for Der p 3, including the third cleavage site of IL-23R, MMR1 and SSTR4, were shown to also be cleaved by Der p 1. Only four out of eight predicted targets for Der p 3 were efficiently cleaved in phage ELISA, whereas, for Der p 6, all eight predicted targets were confirmed and three additional initially predicted for Der p 1 and Der p 3 were shown to also be efficiently cleaved by Der p 6. The sequences, corresponding proteins and cleavage efficiency values (ΔA/A) are represented in Table 3.

### 2.4. Validation of the Interleukin-23 Receptor as a Specific Target for Der p 1 and Der p 6

To confirm the results from the phage display analysis, the specific cleavage(s) of the target(s) identified from PoPS prediction need(s) further validation at the protein level and in a native-like membrane environment. The interleukin-23 receptor (IL-23R), for which two sites cleaved by Der p 1 and Der p 6 were identified, was used as a validation target. IL-23R is a 606-amino acid transmembrane protein with a predicted extracellular domain spanning from residues 24 to 355 that includes the sequence LVWVQ_197_AANA (site 1), VVHVK_163_SLET (site 2) and AVISR_227_AETI (site 3) cleaved by Der p 1 and Der p 6 (site 1) or only Der p 1 (sites 1 and 2), respectively, when displayed in phages.

The full-length rIL-23R, maintained in solution as a proteoliposome, migrated as a 70 kDa single band on SDS-PAGE (Figure 4A,B). When incubated at 37 °C without protease treatment, rIL-23R was stable for at least 5 h but partially degraded after 24 h under our experimental conditions. The degradation profiles of rIL-23R incubated with Der p 1, Der p 3 or Der p 6 were subsequently analyzed at different time points for at least 5 h (Figure 4). rIL-23R was rapidly hydrolyzed by Der p 1 and Der p 6 and the complete degradation was achieved within 30 and 60 min, respectively. In contrast, the migration profile of rIL-23R remained unchanged during at least 5 h of treatment with Der p 3.

Collectively, these data confirm that, in a cell membrane-like environment in which the protein most likely adopts a native topology, the extracellular domain of targets identified in this study based on the specificity profiles of HDM proteases could be efficiently cleaved.

## 3. Discussion

Various experimental approaches correlated the proteolytic activities of allergens with the development of allergic responses in animal models. As examples, active proteolytic cysteine proteases Der p 1 and papain triggered more robust allergic responses compared with their corresponding inactive forms [26,32]. Mice deficient in PAR-2, a receptor activated by numerous serine protease allergens developed less allergen-specific IgE responses and lung inflammation when exposed to HDM [33,34]. Finally, a Der p 1-specific inhibitor could attenuate inflammation triggered by administration of HDM allergen extracts [35]. The human protein substrates targeted by HDM protease allergens, the “HDM protease degradome”, remain under investigated. The task of characterizing these natural protein substrates in humans, although highly challenging, is therefore critical to better understand the impact of the HDM proteases on allergy development.

The present study aimed at predicting the natural substrates of the HDM protease allergens Der p 1, Der p 3 and Der p 6 on a proteome-wide scale. The substrate degradome of Der p 9, a HDM allergen with collagenolytic activity, was not investigated as the production of its recombinant enzymatically active form has proven unsuccessful, while the isolation of natural enzyme requires complex purification protocols as well as large amount of HDM allergen extracts. We deliberately narrowed our search to membrane-associated protein substrates that display at least one extracellular domain, as they represent the first set of cellular proteins exposed to these proteases, notably at the level of the apical side of airway epithelial cells or the dendritic cell surface.

In our approach, peptide libraries displayed on M13 filamentous phage were used for the high throughput profiling of the extended proteolytic specificities of Der p 1, Der p 3 and Der p 6. This approach has already been successfully applied to determine the specificity of various other proteases, including caspases, HIV protease, furin and metalloproteases [31,36,37,38,39]. To our knowledge, with the exception of one report describing combinatorial tetrapeptidic libraries to characterize the Der p 1 specificity for P_1_–P_4_ residues [29], this is the first study which deciphers the extended specificities of HDM proteases towards both P and P′ positions.

In this work, three substrate phage display libraries, X_4_-X-X_4_, X_4_-R/K-X_4_ and X_4_-Y/F-X_4_, were specifically designed to elucidate the extended cleavage patterns of Der p 1, Der p 3 and Der p 6 over the positions P_5_ to P_4_′. The choice of using a fully randomized nonapeptide library X_4_-X-X_4_ for Der p 1 was based on its apparent broad cleavage specificities, which is rather common among papain-like cysteine proteases [29,40]. In contrast, based on previous studies and sequence homologies, we hypothesized that Der p 3 and Der p 6 were enzymatically similar to trypsin and chymotrypsin, respectively [5,7,8,41], which preferentially cleave after Arg/Lys or Tyr/Phe, accordingly. To build robust and unbiased specificity profiles, three selection rounds were performed in each screening campaign, ensuring the profiling of residue preferences at different positions, while keeping a certain level of diversity. The cleavage of X_4_-X-X_4_ library by Der p 1 showed that the protease preferentially hydrolyzed peptide substrates with Val residues at positions P_5_, P_4_ and P_2_, and Lys or Arg at P_1_. Not only are our results in line with those from the microarray analysis of the Der p 1 S_4_–S_1_ subsites [29] but they also match the data describing the P_1_, P_2_, P_3_, and P_4_ specificities of papain, bromelain, human cysteine cathepsins as well as papain-like proteases of parasitic origin, all these proteases preferring Arg and Lys at P_1_ and strictly hydrophobic amino acids at P_2_ [40]. Moreover, Val residue at position P_2_ was preferred for cathepsin B and S [40]. Remarkably, we also highlighted for the first time the importance for Der p 1 of P′ positions, with a marked preference for Ala or Ser residues at P_1_′ and P_2_′, Ala, Arg or Leu in P_3_′ and Val in P_4_′. This extended specificity for Der p 1 is in agreement with the P_2_ to P_2_′ sequences of already described natural substrates for this protease such as α-1-proteinase inhibitor [42], the C-type lectin domain family [19] and the proDer p 1 activation sites [43]. It is also consistent with the specificity matrix for Der p 1 positions P_4_ to P_4_′ available in MEROPS database (Available online: http://merops.sanger.ac.uk). The extended specificity covering positions P_5_–P_4_′ could, at first sight, suggest that Der p 1 cleaves a limited set of protein targets. However, neither the predicted targets of this study nor the proteins previously described as Der p 1 substrates displayed its optimal consensus [19]. This observation indicates that specific recognition and cleavage by Der p 1 can be achieved even if not all of the preferred residues are present. Interestingly, similar conflicting results were observed in a report characterizing the extended cleavage specificity of human thrombin, which showed that the cleaved sequence in its natural substrates displays only 1–30% of the optimal cleavage specificity [44]. Moreover, in addition to specificity requirements, the structure and accessibility of the target sequences as well as the importance of non-active site interaction, exosites and local environmental conditions such as pH, should not be neglected [45,46].

Using the X_4_-R/K-X_4_ phage library, it was shown that Der p 3 preferentially cleaved peptides not only with Arg residue at P_1_, but also with Ala, Ser or Thr at P_1_′. Similar P_1_–P_1_′ specificity was evidenced for human trypsin using phage inhibitors derived from bovine pancreatic trypsin inhibitor (BPTI) [47]. Moreover, our results are in line with those obtained for Sar s 3, a Der p 3 homolog from the scabies mite *Sarcoptes scabiei*, which showed a preference for substrates containing the sequence Arg-Ser(Gly/Ala) at positions P_1_–P_2_′ [48]. Furthermore, similarities were observed between the Der p 3 consensus cleavage site determined by phage display and the targeted sequence of PAR-2, its only natural substrate identified so far. The specificities of Der p 1 and Der p 3 were shown to partially overlap at positions P_2_–P_1_–P_1_′, which is in agreement with data generated with tetrapeptidic fluorogenic substrates and may explain why substrate predicted for Der p 3 can be efficiently cleaved by Der p 1 [29,30]. Der p 6 exhibited strong specificity for Ala at P_2_′, as highlighted by the screening of the X_4_-Y/F-X_4_ library. A clear preference for a Phe over a Tyr residue at P_1_ and for a Ser or a Thr at P_1_′ was also shown.

With the help of the PoPS bioinformatics tool, the identified HDM protease specificities allowed the prediction of human substrates, with particular attention paid to cell surface proteins involved in inflammatory or anti-inflammatory pathways and containing at least one ectodomain. Twenty-one potential targets of Der p 1, Der p 3 and Der p 6 were identified (Table 2). Among them, for the reasons evoked above, with the exception of DC-SIGN experimentally shown to be cleaved by Der p 1, none of the already known protein substrates of these protease allergens were listed [19]. Nevertheless, most of the predicted cleavage sites were efficiently cleaved using phage ELISA.

Interestingly, out of these 21 potential targets, the cleavage of consensus sequences in IL-10R as well as IL-12R by Der p 3 and Der p 1, respectively, could uncover new mechanisms to trigger Th2-biased HDM allergic response through down-regulation of tolerance and Th1 differentiation, the biological activities of IL-12 and IL-10 being critical for such anti-allergic immunity [49,50]. The identification of potential cleavage sites on several receptors from inflammatory pathways including IL-1RII, IL-17R, IL-18R or IL-23Ra as well as IL-3R, a receptor involved in the priming of basophils, may seem more puzzling. Indeed, we could speculate that the cleavages of these receptors by HDM protease allergens could be detrimental in the context of the development of the HDM allergic response. Interestingly, a recent paper showed that cysteine protease antigens such as papain or bromelain cleave the α subunit of murine IL-3 receptor on basophils [51].

Whereas all these predicted cleavages need to be confirmed in the context of cell-expressed receptors, our results could show that HDM allergen proteases do not make a distinction between “Pro-Th2” and “Anti-Th2” protein targets. Moreover, the down-regulation of inflammatory pathways by HDM protease allergens could represent a new mechanism for the tight control of the HDM allergic response. It is, however, evident that the balance between pro-Th2 and anti-Th2 effects of the HDM protease allergens is dependent on the in vivo expression level of these predicted protein substrates, which could vary greatly, in particular according to the allergic status of individuals. [52]. The proportion of the different proteases during the HDM sensitization could also have an impact on the overall degradome of HDM proteases. Of note, cysteine proteases have previously been shown to represent the most important contributors of the protease activity in HDM extracts [53].

To validate our strategy for the identification of new potential protein substrates for Der p 1, Der p 3 or Der p 6, we used recombinant IL-23R embedded in liposomes. We selected this target as three cleavage sites, two for Der p 1 and one for Der p 6, were predicted to be present on this receptor. Moreover, the treatment with Der p 3 could be considered as negative control according to the absence of Der p 3 consensus site in this extracellular domain of this target. The degradation profile of liposome-presented rIL-23R matched the results obtained with phages exposing the corresponding IL-23R cleavage sites, demonstrating that IL-23R represents a new potential target for Der p 1 and Der p 6 but not Der p 3.

## 4. Materials and Methods

### 4.1. Expression, Purification and Maturation of Recombinant Der p 1, Der p 3 and Der p 6

The recombinant zymogen forms proDer p 1, proDer p 3 and proDer p 6 were expressed in the yeast *Pichia pastoris*, purified and then activated into mature proteases as previously described [5,7,43].

### 4.2 Construction of Phage Substrate Libraries

Three phage substrate libraries were created using the fd-Tet-DOG 1 vector based on previously described protocols [37,38,54]. These libraries displayed randomized nonameric peptides at the N-terminus of fd phage coat protein pIII according to the general sequences (positions P_5_–P_4_′): X_4_-X-X_4_, X_4_-R/K-X_4_ and X_4_-Y/F-X_4_, where X represents any amino acid, R/K and Y/F are arginine or lysine and tyrosine or phenylalanine in equivalent ratios, respectively. The randomized sequences were flanked by GPGG spacers to prevent secondary structure formation and preceded by N-terminal polyhistidine tag for phage particle capture and immobilization on nickel (II)-nitrilotriacetic acid (Ni-NTA),) magnetic agarose beads (Qiagen GmbH, Hilden, Germany) [31,37,55]. Three partially degenerated oligonucleotides were designed for the X_4_-X-X_4,_ X_4_-R/K-X_4_ and X_4_-W/F-X_4_ libraries, respectively: 5′-TATTCTCACA*GTGCAC*ATCATCACCACCATCACGGTCCGGGTGGT-(NNK)_9_-GGTGGTCCGGGTCGG*GCGGCCGC*AGAAACTTGTTG-3′ (where N represents any nucleotide and K represents G/T), 5′-TATTCTCACA*GTGCAC*ATCATCACCACCATCACGGTCCGGGTGGT-(NNK)_4_-ARG-(NNK)_4_-GGTGGTCCGGGTCGG*GCGGCCGC*AGAAACTTGTTG-3′ (where R represents A/G) and 5′-TATTCTCACA*GTGCAC*ATCATCACCAC-CATCACGGTCCGGGTGGT-(NNK)_4_-TWT-(NNK)_4_-GGTGGTCCGGGTCGG*GCGGCCGC*AGAAACTTGTTG-3′ (where W represents A/T). These oligonucleotides were annealed to a short complementary primer 5′-CAACAAGTTTCT*GCGGCCGC*-3′, treated with the large Klenow fragment to generate double-stranded DNA, double-digested with restriction endonucleases *ApaLI* and *NotI* (Italic) (NEB, Ipswich, SFK, UK). The restricted DNA fragments were ligated into the similarly digested and dephosphorylated fd-Tet-DOG vector. The final constructions were used for transformation of electrocompetent *E. coli* TG1 cells and tetracycline-resistant colonies were then selected.

### 4.3. Screening of Phage-Substrate Libraries

Phage libraries were amplified and titrated as previously described [55]. The titers of the X_4_-X-X_4_, X_4_-R/K-X_4_ and X_4_-W/F-X_4_ libraries were 7.1 × 10^6^, 2.3 × 10^6^ and 3 × 10^7^ colony forming units (cfu), respectively. The screening protocol was adapted from [56]. For each library 1 × 10^12^ phages were incubated with 100 µL of Ni-NTA magnetic agarose beads equilibrated in phage immobilization buffer (PBS, 5 mM imidazole, 0.05% Tween 20, pH 7.5) for 1 h at room temperature with gentle agitation. Beads were subsequently washed with 5 mL of phage immobilization buffer and 2 mL of PBS pH 7.5 to remove unbound phages. Immobilized phages were then incubated with Der p 1 (preactivated with 1 mM DTT), Der p 3, Der p 6 or PBS at 37 °C. The elution of phage particles by proteolysis of the nonameric randomized sequences was then stopped by the addition of a protease inhibitor (10 µM of *trans*-Epoxysuccinyl-l-leucylamido (4-guanidino) butane (E-64) (Sigma-Aldrich, Saint-Louis, MO, USA) for Der p 1 and 10 µM of phenylmethanesulfonyl fluoride (PMSF, for Der p 3 and Der p 6) (Sigma-Aldrich). Phages released through peptide proteolysis were recovered from the supernatant and were used to infect new bacterial TG1 cells for the preparation of a sub-library. Subsequently, 1 mL of the amplified phages was used for the following round of selection. Negative and positive controls consisting of PBS or PBS + 200 mM imidazole treatment were performed at each selection step to assess cleavage efficiency and specificity. The enrichment rate (ratio of the number of infectious phages eluted by protease selection to the number of phages released in the control experiment without protease treatment) was monitored to assess the efficiency of the different selection cycles. After three successive selection rounds, 75 randomly selected individual clones were sequenced and aligned.

### 4.4. Determination of Der p 1, Der p 3 and Der p 6 Substrate Specificity Profiles and Prediction of New Protein Substrates among the Human Cell Surface Proteome

Substrate specificity profiles of Der p 1, Der p 3 and Der p 6 were determined over nine positions (from P5 to P4′) based on the peptide sequences from the selected eluted phages [57]. Specificity scores (Δσ) were first calculated for each of the 20 amino acids at each position on the basis of their representativeness in the initial libraries and selected sequences as previously described [36,48,57].

(1) $$∆\sigma =\frac{\mathrm{Obs}\left(x\right)-nP\left(x\right)}{\sqrt{nP\left(x\right)\left[1-P\left(x\right)\right]}}$$

Equation (1): Estimation of the standard deviations (Δσ) related to the probability to find any amino acid at a certain position. Obs(*x*) is the number of times amino acid *x* occurs in the selected sequences, *P*(*x*) is the probability of amino acid *x* occurring in a given library and n is the total number of sequences analyzed.

Related standard deviations Δσ were then normalized to the range −5.0 to +5.0 as required by the PoPS interface [57]. Specificity matrices for Der p 1, Der p 3 and Der p 6 were modeled and used to screen, among the human proteome, new potential cell surface protein substrates targeted by these HDM proteases using the PoPS algorithm (Available online: http://pops.csse.monash.edu.au/home.html) (RefSeq database (NCBI) (2006) [57]. The sequence and topology of each putative target were analyzed using the Uniprot database. Amongst the putative targeted proteins, those located intracellularly as well as membrane proteins with predicted cleavage sites located in transmembrane or intracellular domains were manually excluded for further analysis.

### 4.5. Cleavage Site Validation by Phage ELISA

Sequences encoding the potential cleavage sites of the predicted substrates for the HDM proteases Der p 1, Der p 3 and Der p 6 were cloned into the fd-Tet-DOG phage as described above. Then, 10^10^ phages (200 µL) were prepared and incubated for 30 min at 37 °C in PBS pH 7.5 in the presence or absence of either 40 nM of Der p 1 (preactivated with 1 mM DTT), 2 nM of Der p 3 or 4 nM of Der p 6. The reaction was stopped by the addition of specific inhibitors as described above. Phages were then immobilized for 2 h at 25 °C in 96-well Ni-NTA HisSorb plates (Qiagen). Cleaved phages were washed away using PBS pH 7.5, 0.05% Tween 20, while the residual uncleaved phages were detected by means of an anti-M13 monoclonal antibody conjugated to the horseradish peroxidase (HRP) (GE Heatlthcare, Diegem, Belgium) using 3,3′, 5,5′-tetramethylbenzidine (TMB) as substrate (KPL Inc., Milford, MA, USA). The absorbance at 450 nm monitored for treated and untreated phages was then compared.

### 4.6. In Vitro Cleavage of the Recombinant Interleukin-23 Receptor by HDM Proteases

The full-length human recombinant interleukin-23 receptor (rIL-23R, 10 nM) (Abnova, Jhouzih St. Taipei, Taiwan), embedded in proteoliposomes, was incubated at 37 °C in the presence or absence of Der p 1 (2.5 nM, preactivated with 1 mM DTT), Der p 3 (0.25 nM) or Der p 6 (0.5 nM) in PBS pH 7.5 for different periods of time (from 0 to 24 h). At the appropriate time points, proteolysis was stopped by the addition of protease inhibitors (10 µM E-64 or PMSF). rIL-23R degradation was analyzed by SDS-PAGE and revealed using the Sypro Ruby protein gel stain (Bio-Rad, Temse, Belgium) and by western blot analysis using a rabbit anti-human IL-23R (residues 62–75) monoclonal primary antibody (SAB1104999, dilution 1/1000) (Sigma-Aldrich) and a goat HRP/anti-rabbit IgG monoclonal conjugate as secondary antibody (1706515, dilution 1:3000) revealed using the ECL plus western blotting substrate (Bio-Rad).

## 5. Conclusions

In summary, phage display technology allowed to determine the extended substrate recognition profiles of the HDM protease allergens Der p 1, Der p 3 and Der p 6 from position P_5_ to P_4_′. In addition, the corresponding profiles made possible the identification of a panel of potential novel substrates for these protease allergens. Our results could pave the way to a better understanding of the role of these proteases in the mechanism of the HDM allergic response but also to the design of specific protease inhibitors for future anti-allergic treatments as suggested by previous promising studies [35,58].

## Figures and Tables

**Table 1:** Phage libraries and the corresponding sequences displayed at the surface of phage particles in the libraries X_4_-X-X_4_, X_4_-R/K-X_4_ and X_4_-Y/F-X_4_. P_1_ residue is represented in bold. (His6: His-tag; GPGG: glycine/proline spacer; X: random residue; R: arginine; K: lysine; Y: tyrosine; F: phenylalanine; pIII: capsid protein III).

Protease	Library	Displayed Sequence	P_1_ Residue
Der p 1	X_4_-X-X_4_	His6-GPGG-X_4_-X-X_4_-GGPG-pIII	X
Der p 3	X_4_-R/K-X_4_	His6-GPGG-X_4_-R/K-X_4_-GGPG-pIII	R/K
Der p 6	X_4_-Y/F-X_4_	His6-GPGG-X_4_-Y/F-X_4_-GGPG-pIII	Y/F

**Table 2:** Potential protein substrates predicted within the human cell surface proteome for Der p 1, Der p 3 or Der p 6. The down arrow indicates the position of the predicted peptide bond cleaved. Residues corresponding to the most represented amino acids at a determined position in the specificity models are shown in bold.

Target	Proteases	Predicted Cleavage Site P_5_P_4_P_3_P_2_P_1_↓P_1_′P_2_′P_3_′P_4_′	Cleavage Position	Extracellular Domain	Uniprot
DC-SIGN	Der p 1	L**VVIK**↓**SA**EE	295−296	59−404	Q9NNX6
IL-1RII	Der p 1	P**V**AL**R**↓CPQ**V**	49−50	14−343	P27930
IL-12RBII	Der p 1	A**V**A**V**S↓**AA**NS	398−399	24−622	Q99665
IL-17RB	Der p 1	KKC**VK**↓**A**GSL	175−176	18−292	Q9NRM6
IL-23R (site 2)	Der p 1	**VV**H**VK**↓**S**LET	163−164	24−355	Q5VWK5
CLEC1A	Der p 1	**V**QN**IK**↓**LA**GS	109−110	74−280	Q8NC01
CD163b	Der p 1	R**V**E**VK**↓H**A**DT	811−812	41−1359	Q9NR16
IL-3RA	Der p 1, Der p 3	L**V**RG**R**↓**SAA**F	187−188	19−305	P26951
IL-4R	Der p 1, Der p 3	H**V**KP**R**↓**A**PGN	124−125	26−232	P24394
IL-10RA	Der p 1, Der p 3	GYRA**R**↓V**R**A**V**	99−100	22−235	Q13651
IL-18R1	Der p 1, Der p 3	IL**V**R**K**↓**A**DMA	315−316	22−319	Q13478
EphRB1	Der p 1, Der p 3	**VV**QV**R**↓**AR**TV	507−508	18−540	P54762
IL-23R (site 1)	Der p 1, Der p 6	L**V**W**V**Q↓**AA**NA	197−198	24−355	Q5VWK5
CD23	Der p 3	QLEE**R**↓**AAR**N	59−60	48−321	P06734
IL-23R (site 3)	Der p 3	A**V**IS**R**↓AETI	227−228	24−355	Q5VWK5
MMR1	Der p 3	PGGR**R**↓**S**SLS	1042−1043	19−1389	P22897
SSTR4	Der p 3	PGDA**R**↓**AA**GM	43−44	1−46	P31391
IL-5RαII	Der p 6	LHK**GF**↓**S**ASV	94−95	21−342	Q01344
IL-17RCII	Der p 6	VVL**SF**↓QAYP	200−201	21−538	Q8NAC3
IL-17RE (site 1)	Der p 6	SFT**G**S↓**SA**YI	47−55	24−454	Q8NRF9
IL-17RE (site 2)	Der p 6	MHAT**F**↓**SA**AW	386−387	24−454	Q8NRF9
CD1b	Der p 6	**R**AQK**F**↓C**A**L**I**	162−163	18−303	P29016
CD109	Der p 6	EDG**SF**↓**SA**FG	974−975	22−1420	Q6YHK3
MST1R	Der p 6	**VV**P**SF**↓**SA**GG	49−50	25−297	Q04912

**Table 3:** HDM protease cleavage efficiency on predicted targets identified within the human cell surface proteome, revealed by phage ELISA. **A**,**B** and **C**: Substrates efficiently cleaved by Der p 1, Der p 3 and Der p 6, respectively; the targeted cleavage sites and the cleavage efficiencies (ΔA/A) are indicated. The predicted substrates cleaved with (ΔA/A) values greater than 10% are shown in bold. The sequences not predicted but efficiently cleaved are shown in italic. The down arrow indicates the position of the predicted peptide bond cleaved.

**(A)**			
**Target**	**Cleavage Site for Der p 1**	**ΔA/A (%)**	**Shared**
**IL-12RBII**	**AVAVS↓AANS**	**90.9 ± 1.4**	–
***SSTR4***	***PGDAR↓AAGM***	***73.4 ± 1.1***	–
**IL-1RII**	**PVALR↓CPQV**	**65.0 ± 1.8**	**Der p 6**
**IL-23R (site 2)**	**VVHVK↓SLET**	**62.8 ± 12.7**	**–**
**EphRB1**	**VVQVR↓ARTV**	**61.1 ± 6.7**	**Der p 3**
**IL-23R (site 1)**	**LVWVQ↓AANA**	**43.1 ± 2.2**	**Der p 6**
***MMR1***	***PGGRR* ↓*SSLS***	***31.6 ± 1.3***	–
**IL-3RA**	**LVRGR↓SAAF**	**21.5 ± 15.5**	**Der p 3**
**IL-10RA**	**GYRAR↓VRAV**	**17.9 ± 11.3**	**Der p 3**
CLEC1A	VQNIK↓LAGS	6.1 ± 2.12	–
IL-4R	HVKPR↓APGN	5.5 ± 0.5	–
IL-17RB	KKCVK↓AGSL	5.2 ± 2.5	–
IL-18R1	FILVRK↓ADMA	4.9 ± 4.52	Der p 3
DC-SIGN	LVVIK↓SAEE	4.2 ± 1.9	–
*IL-23R (site 3)*	*AVISR↓AETI*	*3.8 ± 0.3*	Der p 6
Ctrl neg	GGSGGSGGS	*1.15 ± 0.1*	–
**(B)**			
**Target**	**Cleavage Site for Der p 3**	**ΔA/A (%)**	**Shared**
**IL-3RA**	**LVRGR↓SAAF**	**83.8 ± 0.2**	**Der p 1**
**IL-18R1**	**FILVRK↓ADMA**	**70.0 ± 9**	**Der p 1**
**EphRB1**	**VVQVR↓ARTV**	**67.2 ± 1.7**	**Der p 1**
**IL-10RA**	**GYRAR↓VRAV**	**54.0 ± 8.0**	**Der p 1**
Ctrl neg	GGSGGSGGS	*3.0 ± 0.6*	–
**(C)**			
**Target**	**Cleavage Site for Der p 6**	**ΔA/A (%)**	**Shared**
**CD109**	**EDGSF↓SAFG**	**94.1 ± 0.1**	**–**
**IL-17RE (site 2)**	**MHATF↓SAAW**	**87.9 ± 0.1**	**–**
**IL-17RE (site 1)**	**SFTGS↓SAYI**	**84.1 ± 1.2**	**–**
**IL-23R (site 1)**	**LVWVQ↓AANA**	**69.4 ± 2.3**	**Der p 1**
**CD1b**	**RAQKF↓CALI**	**30.4 ± 2.0**	**–**
*IL-12RBII*	*AVAVS↓AANS*	*4.7 ± 0.6*	*–*
MST1R	VVPSF↓SAGG	4.3 ± 2.1	–
*IL-1RII*	*PVALR↓CPQV*	*2.9 ± 1.1*	Der p 1
IL-5RαII	LHKGF↓SASV	2.5 ± 0.8	–
IL-17RCII	VVLSF↓QAYP	2.4 ± 0.2	–
*IL-23R (site 3)*	*AVISR↓AETI*	*1.9 ± 0.4*	Der p 1
Ctrl neg	GGSGGSGGS	*0.0 ± 0.0*	–

## Supplementary Materials

Supplementary materials can be found at www.mdpi.com/1422-0067/18/7/1373/s1.

## Abbreviations

CD1b	T-cell surface glycoprotein CD1b
CD23	Low-affinity immunoglobulin epsilon Fc receptor
CD109	150 kDa TGF-β-1-binding protein CD109 antigen
CD163b	Scavenger receptor cysteine-rich type 1 protein M160
CLEC1A	C-type lectin domain family 1 member A
DC-SIGN	Dendritic cell-specific
EphRB1	Ephrin type B receptor 1
HRP	Horseradish peroxidase. ICAM-3-grabbing non-integrin 1
IL	Interleukin
R	Receptor
A	α
B	β
II	Type 2
MMR1	Macrophage mannose receptor 1
MST1R	Macrophage-stimulating protein receptor
SSTR4	Somatostatin receptor type 4
