# Supplementary material for: Profiling the Extended Cleavage Specificity of the House Dust Mite Protease Allergens Der p 1, Der p 3 and Der p 6 for the Prediction of New Cell Surface Protein Substrates
Source: Int J Mol Sci. 2017 Jun 27;18(7):1373. doi: 10.3390/ijms18071373 (PMC5535866; doi:10.3390/ijms18071373)
Supplement: Supplementary file 1 [file ijms-18-01373-s001.pdf]

Table S1. List of the potential substrates of Der p 1, Der p 6 and Der p 9 as identified among the human proteome using the PoPS algorithm. Proteins displaying at least one extracellular site susceptible to proteolysis were selected among the 500 best hits during the PoPS analysis (threshold score=10). Hypothetical and predicted proteins were not considered. Attention was paid to proteins directly or indirectly involved in allergy- or inflammation-related immune mechanisms on the basis of experimental findings (represented in bold).

Potential Substrates	Targeted Sequences in the Extracellular Region	Predicted Proteases
150 kDa TGFβ1-binding protein CD109 antigen	EDGSFSAFG	Der p 6
Artemin	LVPVRALGL	Der p 1
Butyrophilin like protein 9	SVVVRAGAL	Der p 1, Der p 3
CD C-type lectin-like receptor 1	VQNIKLAGS	Der p 1
Collagen α-1(XII) chain	FVKVRAFLE	Der p 3
Collagen α-1(XIV) chain	RDTLFTAES	Der p 6
C-type lectin domain family 14 member A	LSTVRAGAE	Der p 1
Dendritic cell specific ICAM-3-grabbing non-integrin 1	LVVIKSAEE	Der p 1
Ephrin type B receptor 1	VVQVRARTV	Der p 1, Der p 3
Fc receptor-like protein 5	LRVPRAQAV	Der p 1
	FRAPRAQAV	Der p 1
G protein-coupled receptor 64	PVVPRATVL	Der p 1
G protein-coupled receptor 112	GDLIRTTSE	Der p 3
G protein-coupled receptor 173	RVFVKACAV	Der p 1
Interleukin-1 receptor type II	PVALRCPQV	Der p 3
Interleukin-3 receptor α	LVRGRSAAF	Der p 1, Der p 3
Interleukin-4 receptor	HVKPRAPGN	Der p 1, Der p 3
Interleukin-5 receptor α type II	LHKGFSASV	Der p 6
Interleukin-10 receptor α	GYRARVRAV	Der p 1, Der p 3
Interleukin-12 receptor β type II	AVAVSAANS	Der p 1
Interleukin-17 receptor B	KKCVKAGSL	Der p 1
Interleukin-17 receptor C type II	VVLSFQAYP	Der p 6
Interleukin-17 receptor E	SFTGSSAYI	Der p 6
	MHATFSAAW	Der p 6
Interleukin-18 receptor 1	FILVRKAIM	Der p 1, Der p 3
Interleukin-23 receptor	LVWVQAANA	Der p 1, Der p 6
	VVHVKSLET	Der p 1
	AVISRAETI	Der p 3
Immunoglobulin DC C subclass member A	PRNVRAVSV	Der p 1
Integrin α-3	IVLLRARPV	Der p 3
Integrin α-9 precursor	VVLLRARPV	Der p 3
Integrin β-5	LYKNFTALI	Der p 6
Junction plakoglobin	VVVTKAAMI	Der p 1, Der p 3
Killer cell immunoglobulin-like receptor	GSMMRALAG	Der p 3
Low affinity immunoglobulin ε Fc receptor (FcεRIIa)	QLEERAARN	Der p 3
Low density lipoprotein-related protein 1B precursor	SVAVFGAYI	Der p 6
	SVDPFEAYI	Der p 6
Macrophage mannose receptor 1 (MMR1)	PGGRRSSLS	Der p 3
Macrophage stimulating protein receptor (MST1R)	VVPSFSAGG	Der p 6
Metalloproteinase inhibitor 2	DVVIRAKAV	Der p 1, Der p 3
Neutrophil elastase preproprotein	NVNVRAVRV	Der p 1, Der p 3
Olfactory receptor 2T8	PVLVRLACA	Der p 3
Pentraxin-related protein PTX3	RLESFSACI	Der p 6
Platelet-derived growth factor receptor α	VVEVRAYPP	Der p 3
Prolow-density lipoprotein receptor-related protein 1	DVHVKAGRV	Der p 1

Prosaposin	VVAPFMANI	Der p 6
Prosaposin-like protein-1	GNRRRARAV	Der p 3
Protocadherin γ-A12	PQASYSAYI	Der p 6
	DNAGYSARA	Der p 6
Scavenger receptor cysteine-rich type 1 protein M160	RVEVKHADT	Der p 1
Somatostatin receptor type 4	PGDARAAGM	Der p 3
Semaphorin-4A	GPMSRSLRP	Der p 3
	GDERRALSF	Der p 3
T-cell surface glycoprotein CD1b	RAQKFCALI	Der p 6

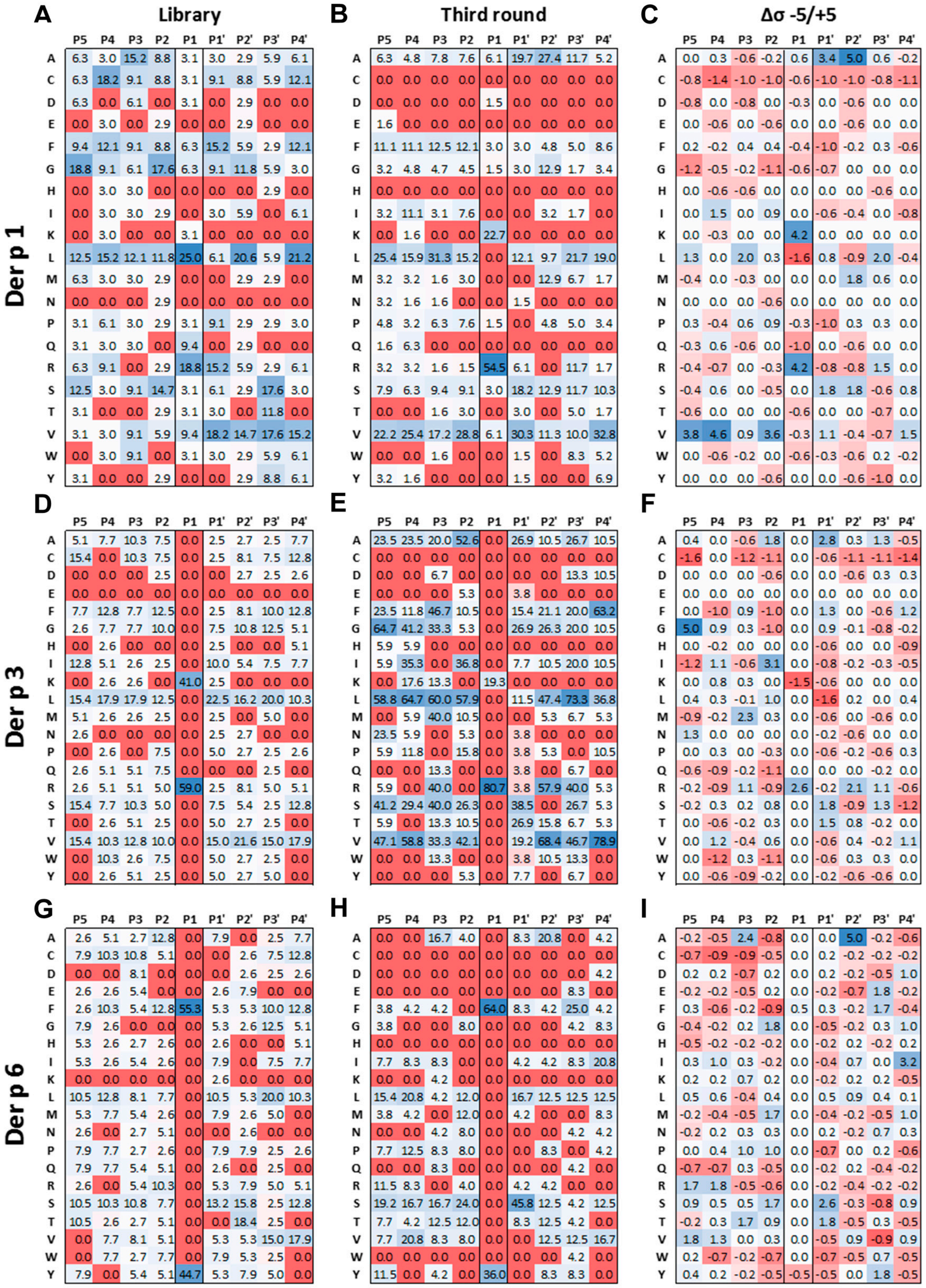

Figure S1. Phage substrate library diversity and extended substrate specificity profile of Der p 1, Der p 3 and Der p 6. Phage substrate library diversity for positions P_5 to P_4' for libraries X_4-X-X_4 (Der p 1), X_4-R/K-X_4 (Der p 3) and F X_4-Y/F-X_4 (De r p 6) before (**A**, **G** and **D**) and after (**B**, **E** and **H**) three rounds of selection with the corresponding protease. Values are expressed as percentage of each amino acid for a specific position. The sequences used to validate the libraries and to establish the specificity models are available upon request. Normalized Δσ specificity matrices (−5 to +5) calculated for each

amino acid at positions P_5 to P_4' after three rounds of selection for libraries (**F**) X_4-X-X_4 with Der p 1, (**F**) X_4-R/K-X_4 with Der p 3 and (**I**) F X_4-Y/F-X_4 with Der p 6.
